# Measuring mental wellness among adolescents living with a physical chronic condition: a systematic review of the mental health and mental well-being instruments

**DOI:** 10.1186/s40359-021-00680-w

**Published:** 2021-11-08

**Authors:** Zaida Orth, Brian van Wyk

**Affiliations:** School of Public Health, University of the Western Cape, Robert Sobukwe Rd, Bellville, 7535 South Africa

**Keywords:** Adolescent, Mental Health, Well-being, Health, Measurement

## Abstract

**Background:**

Globally, promoting mental health and well-being among adolescents has become a public health priority, especially for adolescents living with a physical chronic condition (CC), as research suggests they may be more at risk of developing mental health co-morbidities. Valid and reliable instruments are needed to measure and better understand mental health and mental well-being among adolescents living with a CC. To this end, we reviewed studies reporting on mental health and well-being instruments used in adolescent populations living with a chronic physical condition/disease globally.

**Methods:**

We used a systematic review method guided by PRISMA to identify assess mental health and mental well-being instruments used in adolescents living with a CC. In this instance, mental health instruments were defined as those representing negative domains of mental health (i.e. depression and anxiety) while mental well-being instruments included positive aspects of mental health (i.e. self-concept and resilience).

**Results:**

We identified 22 articles, which included 31 instruments that were used to measure either mental health (n = 8) or mental well-being (n = 15) or both (n = 8) in adolescents living with a CC. Of these, thirteen studies used a Health-Related Quality of Life (HRQoL) scale to measure mental health and/or mental well-being. The KIDSCREEN questionnaires and the Strengths and Difficulties Questionnaire were identified as being frequently used across the 22 studies. Additionally, 7 out of the 31 instruments were disease specific, with 3 focusing on adolescents with diabetes. All the instruments were developed in high income countries and adapted for use in lower- and middle-income countries (LMICs). Adolescents with Type 1 Diabetes (n = 7) and HIV (n = 4) were researched in 11 out of 22 studies. Only eight studies were conducted in LMIC, of which four were in Africa.

**Conclusions:**

HRQoL instruments are useful in measuring mental health and well-being in adolescents living with a CC. However, relatively few valid measures of mental health and mental well-being for adolescents living with a CC exist, which accentuates the paucity of research on mental health and mental well-being of adolescents who are living with a CC. Specific measures need to be developed in and for LMICs where cultural contexts affect mental well-being in unique ways.

*Systematic review registration*: PROSPERO CRD42020186707.

## Background

As of 2015, there were an estimated 1.2 billion adolescents (aged 10–19 years), representing 16 per cent of the global population—making them the largest group of adolescents in history [1, 2]. In recent years, the global public health agenda has shifted to recognise the important role adolescent mental health plays in achieving global development goals [1, 2]. According to the World Health Organisation (WHO) [2], mental health conditions account for 16% of the global burden of disease in adolescents. Depression, anxiety, self-harm and childhood behavioural disorders have been reported as the leading causes of disability and illness [3]. It is further argued that half of all chronic mental disorders will start during adolescence, with approximately 75% of adults reporting onset of a mental health problem before the age of 24 years [4]. However, the majority of adolescent mental health problems often go undiagnosed and untreated [5]. Crenna-Jennings and Hutchinson (2020), for example, report that despite the increased investment in child and adolescent mental health services (CAHMS) in England, considerable treatment gaps persist as evidenced in approximately one quarter of children and adolescents referred to mental health specialists not receiving treatment [6]. This is concerning as adolescence represents a crucial period of development, where exposures, learnt behaviours and experiences can set the trajectory for an individual’s mental and physical health in adult life [5, 7].

Concomitantly, children and adolescents with physical chronic conditions (CC) are at increased risk for developing mental health problems or co-morbidities [8, 9]. Alderman et al. [10] confirmed a global trend of increasing number of paediatric patients living with chronic medical conditions. In 2017, Jin et al. [11] reported that the overall prevalence of CCs among child and adolescent populations is estimated at 15–20%. According to Sawyer [10], initial stresses associated with diagnosis, ongoing stresses from treatments, social disruption, perceived stigma, marginalisation, and changes in plans and expectations about the future present substantive challenges to the social and emotional well-being of adolescents living with a CC [10]. While most child and adolescent CC are not preventable by lifestyle changes, it is possible to prevent or modify the socially mediated co-morbidities that are experienced by adolescents living with CC [9]. However, there are various challenges as reports indicate that adolescents living with CC experience various attitudinal, stigma-related and structural barriers to accessing mental health services as well as psycho-social support [12]. Furthermore, the overall mental well-being of chronically ill adolescents is largely determined by, among others, the severity of the disease, the amount of treatment required, and the psychological and social complications associated with such conditions [12, 13].

Given the link between adult and adolescent health, it is necessary to promote a life-course perspective in adolescent health which advocates for effective interventions during adolescence to protect public health investments in child survival and early childhood development, and to ensure the physical and mental health and healthy development of the next generation [14]. Glasner suggests that almost 70% of disease burden in adults can be prevented through early interventions during adolescence [3]. However, it is argued that the preventative strategies to reduce the effect of mental health problems need to go beyond the traditional disease model of mental health. To this end, it is imperative to widen the focus from providing care and treatment for adolescents diagnosed with a mental health disorder to include those who experience challenges to their mental health and well-being before diagnoses are made.

In line with the United Nations (UN) Sustainable Developmental Goal 3 (SDG 3)—which aims to promote well-being for all ages—many countries and organisations are aiming to improve the development of age-appropriate interventions to provide psychosocial support and services to adolescents [15, 16]. Despite the increased focus on adolescent mental health on the global health agenda, there is a lack of evidence concerning mental health conditions among adolescents, especially in LMICs [17]. To address this, UNICEF has launched a project to develop a measure to determine prevalence of mental illness among adolescents at the population level [5] to inform policy makers and healthcare workers, and guide intervention and treatment programmes. Current instruments in use to measure mental health are based on the traditional clinical psychology definitions of mental health as a pathology, which focuses on psychiatric disorders, general mental health disorders, emotional and behavioural problems, psychological distress and lower levels of illness symptoms as representative of mental health [2, 18]. As such, mental health has popularly been used as a euphemism for ‘mental illness’ [19].

However, it has been argued that mental health is more than the absence of illness, therefore, instruments measuring general mental health should also make provisions to include a high degree of psychological well-being [20, 21]. Mental health should then include a focus on the presence of wellness and what it means for an individual to flourish. In contrast to the pathological view of health, positive psychologists have shifted their views to focus on positive mental health or psychological well-being (mental wellness) [19]. From the above-mentioned perspective, mental health is viewed as including both hedonic (feeling well) and eudemonic (functioning well) traditions of well-being [22].

Research on well-being in mental health has gained significant interest as evidence suggests that positive mental health aids as a recovery factor as well as a protective factor against pathology, including both physical and mental [16, 20, 21]. Measures of mental well-being are useful in assessing the strengths and resilience that adolescents possess which in turn in essential to promote positive mental health (wellness) and youth development [23]. However, there is a lack of studies focused on the effectiveness of such measures or on identifying which mental well-being domains are the most useful for screening and assessment [23]. Considerations should be made that focus specifically on adolescent mental health and its association with physical health, especially given the rise of CCs.

This paper reports on a systematic review of mental health and well-being instruments used in adolescent populations living with a chronic physical condition/disease globally.

## Methods

The current review is based on a larger systematic review of mental health instruments for adolescents [3]. For the purpose of this paper, we have chosen to focus on instrument used specifically for adolescents living with a physical CC. For the purpose of this study, general mental health and well-being instruments are those that measure ‘generic’ outcome measure that does not aim to diagnose and can be applied in a wide range of settings [24]. In other words, these well-being and general mental health factors may include social and psychological functioning, relationships with others, social support, self-perception, and quality of life. The seven steps described by Eggar, Davey and Smith [25] were used to guide the review process, namely: (1) *formulate the review question;* (2) *define the inclusion and exclusion criteria*; (3) *develop a search strategy*; (4) *study selection*; (5) *assess the quality of studies*; (6) *extract data*; and (7) *analyse or synthesis the data*.

### Review question

We identified the following research question

1. What instruments are used to measure the mental health and well-being of adolescents living with a chronic physical condition/disease?

### Inclusion and exclusion criteria

The inclusion criteria for the search are as follows:

1. published in peer reviewed journals or grey literature;
2. the sample includes adolescents between the ages of 10–19 years;
3. the measure used was a self-report measure of general mental health and/or well-being;
4. quantitative and mixed methods studies;
5. Studies aimed at developing or validating instruments [3].

Studies will be excluded based on the following criteria:

1. Review papers or case studies;
2. Screening tools for mental disorders or measures that are disorder/symptom specific [3].

The decision to include studies with adolescent samples between the ages of 10–19 years is based on the WHO definition of adolescents [3]. The aim of this study is to review instruments used with adolescents specifically and that recognise adolescence as a unique period of development. Studies that focused on adults or young adults, where 18–19-year-old adolescents were included in the sample were therefore excluded [3]. Furthermore, studies with measures aimed at diagnosing mental health disorders, or that are specific to mental illness were excluded [3]. As such, we do not consider adolescents who have been diagnosed with a chronic mental illness/disorder. While studies indicate that adolescents with a physical CC may have mental illness diagnoses as co-morbidities, our interest lies in identifying instruments which may be used to measure general health among adolescents with a physical CC, which can be used to inform mental health services and intervention to prevent mental health problems from developing into mental illness co-morbidities.

### Search strategy

The search strategy was developed in consultation with the university’s community and health sciences faculty librarian. The search strategy was broad to include all research articles that use a psychological or psychometric instrument to measure mental health outcomes among adolescents [3]. A systematic database search was performed using Ebscohost (Psycharticles, Academic Search Premier), Cumulative Index of Nursing and Allied Health Literature (CINAHL), Educational Resource Information Center (ERIC), Medical Literature Analysis Retrieval System Online (MEDLINE) and Sabinet. Full-texts searches were done using the following key words for the search strategy; “((adolescen* OR teenage* OR young people OR youth) [AND] (psychological instrument OR measure* OR tool) [AND] (mental health OR mental well-being OR psychological well-being) [AND] {psychometri*; reliability*; validit*)) [3, 24].

### Study selection

Studies were included in the systematic review using the PICOT mnemonics for reviews (Table 1).

**Table 1:** PICOT

Patient population	Adolescents aged 10–19 years
Intervention of Interest	Measure general mental health and/or well-being among adolescents living with a chronic physical condition/disease
Comparison interventions	Not applicable
Outcomes	Mental health and psychological well-being
Time	2000–2020
Other considerations	Study designs: Quantitative method or mixed methods Language: All

The time period of the search strategy was chosen due to the paucity of research in this area [2, 17, 26]. Furthermore, the prioritization of adolescent health and the focus on adolescent friendly services occurred after 2000 [27]. The screening and reporting of the review was conducted in accordance with the Preferred Reporting Items for Systematic Reviews and Meta-Analyses (PRISMA) guidelines. The number of hits for each database was recorded and the citations were exported to Mendeley citation software. Following this, two reviewers (ZO & FM) independently reviewed all the titles and abstracts to assess which articles are appropriate for inclusion. The full-text articles of the included abstracts were downloaded and independently reviewed to determine which articles should be included for the final assessment [25, 28].

### Quality assessment

Each of the potentially relevant articles included in the review was evaluated using the SFS scoring system (version D), which is an appropriate tool to use for assessing the quality studies in this review as it allowed the two reviewers to assess the appropriateness of the methodological elements of the included studies, such as the psychometric properties of the instruments and the theoretical and operational definitions used to define constructs [28]. The SFS version D scoring systems contains 29 questions covering the following sub-sections, namely: (1) *purpose of the measure;* (2) *methodological rigour*; and (3) *general considerations*. The overall quality of the study is based on the score as weak (0–25%), moderate (26–50%), strong (51–75%), or excellent (76–100%). Only articles with a score of 51% and above were included in the synthesis.

### Data extraction and synthesis

A descriptive meta-synthesis approach was used to identify and describe the mental health instruments used among adolescent populations. The synthesis of information regarding each instrument was presented in tabular form to display relevant information [29]. The article information was entered into an excel sheet and the sample characteristics (ages, gender, school grade, etc.) geographic location, physical health, mental health and well-being domains and purpose of the instrument were extracted. For the purpose of this study, only data presented in the articles will be used as we are interested in how the data is reported.

### Ethics

Ethics approval is not required as the systematic review does not involve the participation of human subjects; rather it involves reviewing and collecting data from publicly available sources. However, this review forms part of the first-author’s doctoral research project which received ethical clearance from the University of the Western Cape Biomedical Research Ethics committee (BM19/09/18).

## Results

**Fig. 1:**
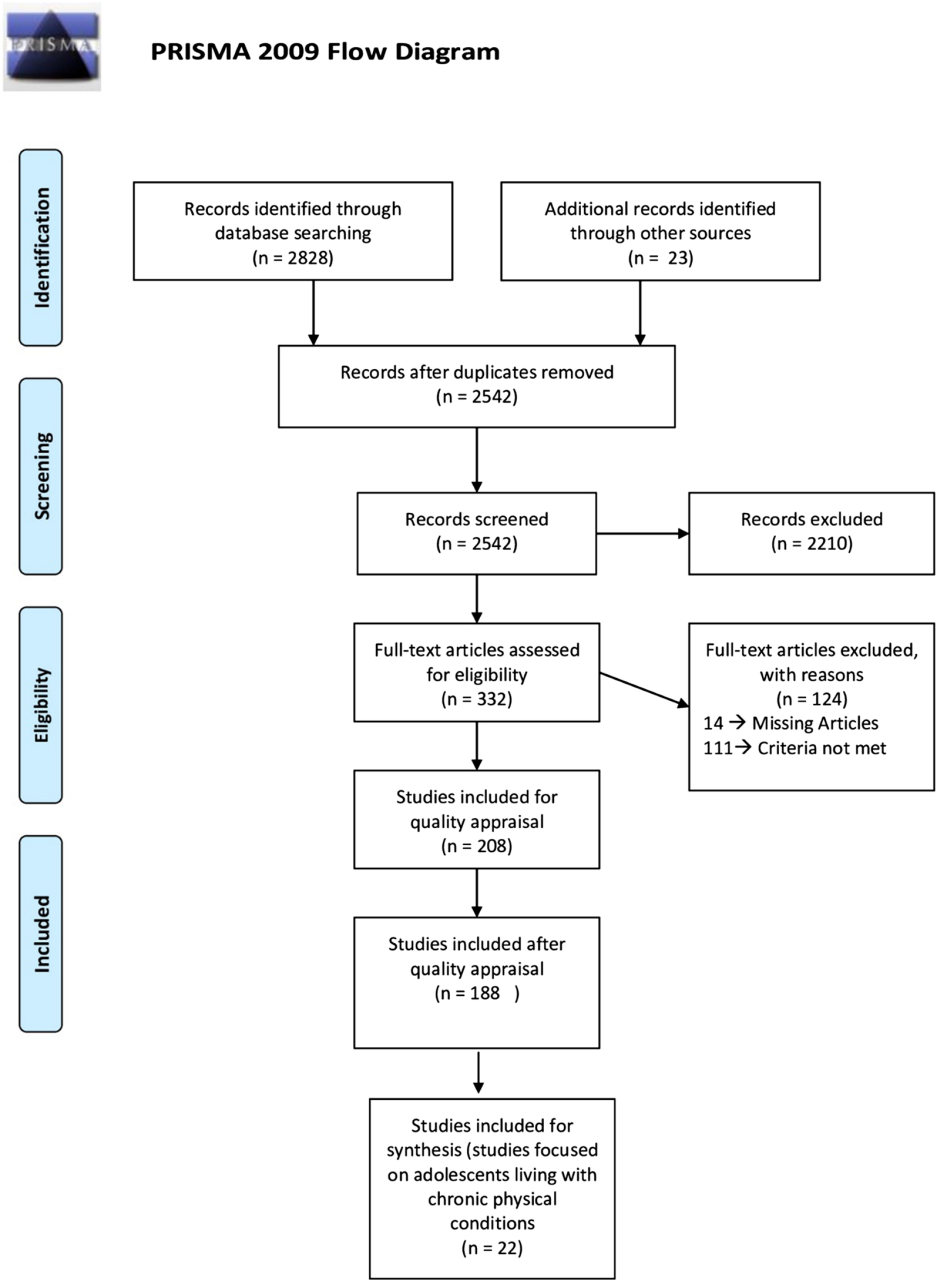
PRISMA diagram

In accordance with PRISMA guidelines, we completed a flowchart detailing the selection process (Fig. 1). Following the screening, we included 208 articles for the quality appraisal. From this, 20 articles scored below 51% on the SFS scoring system and were subsequently excluded, leaving a sample of 188 articles. We screened the full text of 188 eligible articles and identified 22 articles which included samples of adolescents living with a CC or disease.

### Study characteristics

An overview of the study characteristics is presented in Table 2. The sample sizes in the studies ranged from 49 to 1938, and the participants ranged from 8 to 19 years. More than half of the studies were conducted in developed countries: three in Netherlands, and two each in Canada, United States of America, Australia, and Poland, and one each in France, Taiwan, Spain and Germany.

**Table 2:** Characteristics of included studies (N = 22)

References	Country and setting	Sample size	Age range (in years)	Chronic condition	Instruments
Boyes et al. [30]	South Africa, Eastern Cape	1060	10–19	HIV	Child Depression Inventory—Short Form (translated to Xhosa) Revised Children’s Manifest Anxiety Scale (translated to Xhosa)
Cavasos-Rehg et al. [31]	Uganda, Southwest Uganda	702	10–16	HIV	Beck Hopelessness scale Children's Depression Inventory Tennessee Self-Concept Scale (All three instruments were adapted to be culturally appropriate in the Ugandan context)
Chen et al. [32]	Taiwan	500	15–20	Congenital Heart Disease	Healthcare Needs Scale for Youth with Congenital Heart Disease (Mandarin) The questionnaire on health needs for adolescents (Mandarin) WHO Quality of Life-BREF (Taiwan version)
Cox et al. [33]	United States of America, Wisconsin	195	8–17	Asthma, Diabetes, Sickle cell disease	Patient-Reported Outcomes Measurement Information System (PROMIS)
Davis et al. [34]	Australia, Melbourne & Queensland	87	12–18	Cerebral Palsy	Cerebral Palsy Quality of Life Questionnaire-Teen KIDSCREEN-10 Paediatric Quality of Life Inventory
De Alvegera et al. [35]	Brazil, São Paulo	212	12–17	Chronic Illness (cancer, type 1 diabetes, cystic fibrosis)	The FACIT-Sp-12 Spiritual Well-Being Scale (Translated to Portuguese)
De Wit et al. [36]	Netherlands, Rotterdam & Amsterdam	84	8–18	Type 1 Diabetes	Monitoring Individual Needs in Diabetes Youth Questionnaire (MY-Q) (Dutch version) Paediatric Quality of Life Inventory (Dutch version) WHO-5 wellbeing index (Dutch version)
De Wit et al. [37]	Netherlands, North Holland	91	13–17	Type 1 Diabetes	WHO-5 wellbeing index (Dutch version) Center for Epidemiologic Studies Depression Scale (CES-D) (Dutch version) Child Health Questionnaire (CHQ-CF87) (Dutch version)s
Gentz et al. [38]	Namibia, Windhoek	99	12–18	HIV	Strengths and Difficulties Questionnaire (Oshimwaba and English versions)
Glowacki et al. [39]	Poland, Poznan	36	10–17	Adolescent Idiopathic Scoliosis	Strengths and Difficulties Questionnaire (Polish version)
Goldbeck et al. [40]	Germany, Southern Germany	70	16–38	Cystic Fibrosis	The Short Form 36 Health Survey (SF-36) (German version) The Quality of Life Profile for Chronic Diseases (PLC) (German) The Questions on Life Satisfaction (FLZ) (German)
Kaunda-Khangamwa et al. [41]	Malawi, Blantyre	406	15–19	HIV	Child Youth Resilience Measurement (CYRM-28) (translated to Chicewa)
Klages et al. [42]	United States of America, Tennesee	181	12–18	Diabetes	Diabetes Stress Questionnaire The Pediatric Quality of Life Inventory 3.2 Diabetes module
Mayoral et al. [43]	Spain, Barcelona	136	8–19	Type 1 Diabetes	EQ-5D-Y (Spanish version) KIDSCREEN-27 (Spanish version) Strengths and Difficulties Questionnaire (Spanish version)
Misterka et al. [44]	Poland, Poznan	52	11–18	Juvenile Idiopathic Arthritis	Strength and Difficulties Questionnaire (Polish version)
Pavlova et al. [45]	Canada, Alberta	147	8–18	Chronic Pain	Patient-Reported Outcomes Measurement Information System (PROMIS)
Power et al. [46]	Bangladesh, Sirajganj district	154	10–18	Cerebral Palsy	Cerebral Palsy Quality of Life Questionnaire-Teen (translated to Bengali) Bengali version Kidscreen-27 Bengali version Strengths and difficulties questionnaire
Ramirez-Hernandez et al. [47]	Mexico, Mexico City	71	8–18	Strabismus	Kidscreen-52 Spanish version
Rohenkal et al. [48]	Netherlands, Hilversum	49	8–18	Short Stature	Quality of Life in Short Stature Youth (QoLISSY) (translated to Dutch) KIDSCREEN-52 (Dutch version)
Sapin et al. [49]	France	1938	10–17	180 inpatient youth (asthma & diabetes) 254 chronic disease	Vécu et Santé Perçue des Adolescents (VSP-A) (French)
Soltani et al. [50]	Canada, Alberta	145	8–18	Chronic Pain	Patient-Reported Outcomes Measurement Information System (PROMIS) The Paediatric Quality of Life Inventory (Peds-QL)
Szyndler et al. [51]	Australia, Sydney	52	12–18	Cystic Fibrosis	The Cystic Fibrosis Questionnaire (CFQ) The Hunter Opinions and Personal Expectations Scale (HOPES)

Most studies (n = 18) were published after 2010, with only 4 studies published before 2010. Adolescents with Type 1 Diabetes (n = 7) [33, 35–37, 42, 43, 49] and HIV (n = 4) [30, 31, 38, 41] constituted half of the total number of studies. Other conditions were Asthma [33, 49] Cerebral Palsy [34, 46], Cystic Fibrosis [35, 40, 51] and Chronic Pain [45, 50] with two studies each: with single studies on Congenital Heart Disease [32], Sickle cell disease [33], Cancer [35], Adolescent Idiopathic Scoliosis [39], Juvenile Idiopathic Arthritis [44], Strabismus [47] and Short Stature [48], and Chronic Disease (unspecified by authors) [49]. Additionally, 3 of the studies [33, 35, 49] used samples of adolescents living with a various chronic illness, rather than looking at adolescents with a specific chronic illness, thereby suggesting that the instruments used in these studies were not symptom/disease specific. All four studies that were conducted in Africa involved ALHIV [30, 31, 38, 41] while studies involving adolescents living with type 1 diabetes are mostly from European and American countries [33, 36, 37, 42, 43, 49]. The KIDSCREEN [n = 5] and Strengths and Difficulties Questionnaire [n = 5] were the most frequently used measuring instrument.

### Instruments measuring mental health and mental well-being in adolescents 

From the 22 articles, we identified a total of 31 instruments that were used to measure either mental health (n = 8) or mental well-being (n = 15) or in combination (n = 8) (Table 3). We categorised the mental health instruments as those that measure symptoms or aspects related to a specific mental illness (i.e. symptoms of depression), and mental well-being instruments as those that measure aspects related to [over-all] mental wellness or positive mental health (i.e. resilience, hopefulness).

**Table 3:** Characteristics of the Included Instruments

Instrument	Aim of the Instrument	Subscales in the instrument	# Subscales	Mental health subscales	Mental wellbeing subscales	Physical wellbeing	Studies
Beck Hopelessness scale	Hopelessness	Feelings about the future Loss of motivation Expectations	3	✓ (n = 3)			[31]
Cerebral Palsy Quality of Life Questionnaire-Teen	QoL in adolescents living with Cerebral Palsy	Global QoL, social wellbeing, emotional wellbeing, school wellbeing, physical wellbeing, participation, communication, pain	8		✓ (n = 5)	✓ (n = 3)	[34, 46]
Child Youth Resilience Measurement (CYRM-28)	Measures resilience	Individual, relational, community, cultural	4		✓ (n = 4)		[41]
Cystic Fibrosis Questionnaire (CFQ)	Measures HRQoL in patients with Cystic Fibrosis	Physical, role limitations/ school performance, energy/wellbeing, emotional state, social limitations	5		✓ (n = 2)	✓ (n = 3)	[51]
EQ-5D-Y	Measure HRQoL	Mobility, looking after myself, doing usual activities, having pain or discomfort and feeling worried, sad or unhappy	5	✓ (n = 1)	✓ (n = 1)	✓ (n = 3)	[43]
FACIT-Sp-12 Spiritual Well-Being Scale	Measure Spiritual Wellbeing	Spiritual Well-Being, Meaning, Peace, Faith	4		✓ (n = 4)		[35]
Healthcare Needs Scale for Youth with Congenital Heart Disease (HNS-CHD)	Measure of Healthcare Needs	Health, family, individual, interpersonal and policy needs	5		✓ (n = 2)	✓ (n = 1)	[32]
Hunter Opinions and Personal Expectations Scale (HOPES)	Measure of hope and despair for adolescents and adults	Hope, despair, global personal happiness	3	✓ (n = 1)	✓ (n = 2)		[51]
KIDSCREEN-10	Measure HRQoL in children and adolescents	Unidimensional construct of HRQOL	1		✓ (n = 1)	✓ (n = 1)	[34]
KIDSCREEN-27	Measure HRQoL in children and adolescents	Physical Well-Being, Psychological Well-Being, Autonomy & Parents, Peers & Social Support and School Environment	5		✓ (n = 4)	✓ (n = 1)	[43, 46]
KIDSCREEN-52	Measure HRQoL in children and adolescents	Physical, Psychological Wellbeing, Moods and Emotions, Self-Perception, Autonomy, Parent Relations and Home Life, Social Support and Peers, School Environment (6 items), Social Acceptance (Bullying), Financial Resources	10		✓ (n = 8)	✓ (n = 1)	[47, 48]
Paediatric Quality of Life Inventory (Peds-QL)	Measure HRQoL	Physical Functioning (8 items) Emotional Functioning (5 items) Social Functioning (5 items) School Functioning (5 items)	4		✓ (n = 3)	✓ (n = 1)	[34, 36, 50]
Quality of Life in Short Stature Youth (QoLISSY	Measures QoL	Physical, Social, Emotional, Coping, Treatment, Beliefs	6		✓ (n = 4)	✓ (n = 2)	[48]
Tennessee Self-Concept Scale	Measures self-concept	Physical, moral, personal, family, social, academic	6		✓ (n = 5)	✓ (n = 1)	[31]
Vécu et Santé Perçue des Adolescents (VSP-A)	Measure HRQoL	Vitality, Psychological Well-being, Relationships with Friends, Leisure Activities, Relationships with Parents, Physical Well-being, Relationships with Teachers, School Performance, Body Image and Relationships with Medical Staff	10		✓ (n = 8)	✓ (n = 2)	[49]
WHO-5 wellbeing index	Measures current mental wellbeing	Mental /Emotional wellbeing (unidimensional)	1		✓ (n = 1)		[36, 37]
WHO Quality of Life-BREF	Measures QoL	Physical health, psychological health, social relationship and environment	4		✓ (n = 3)	✓ (n = 1)	[32]
Center for Epidemiological Studies Depression	Symptoms of depression	Depressive affect Somatic complaints Positive affect Interpersonal activity	4	✓ (n = 4)		✓ (n = 1)	[37]
Child Depression Inventory (CDI)	Symptoms of depression	Anhedonia, negative mood/physical symptoms, negative self-esteem, interpersonal problems, Ineffectiveness	5	✓ (n = 5)			[31]
Child Depression Inventory—Short Form	Symptoms of depression	Negative mood/physical symptoms, negative self-esteem, interpersonal problems, ineffectiveness	4	✓ (n = 4)			[30]
Paediatric Quality of Life Inventory Diabetes Module (PedsQL-DMTM)	Measure HRQoL in adolescents with Diabetes	general concerns about diabetes, treatment, worry, and communication	4	✓ (n = 1)	✓ (n = 1)	✓ (n = 2)	[42]
Revised Children’s Manifest Anxiety Scale	Measures symptoms of anxiety in children and adolescents	Psychological anxiety, worry/oversensitivity, social concerns/concentration	3	✓ (n = 3)			[30]
Strengths and Difficulties Questionnaire (SDQ)	Emotional and Behavioural Screening Questionnaire	Emotional symptoms subscale Conduct problems subscale Hyperactivity/inattention subscale Peer relationships problem subscale Prosocial behaviour subscale	5	✓ (n = 5)			[38, 39, 43, 44, 46]
The questionnaire on health needs for adolescents	Measure of Healthcare Needs	physical health, reproductive health, mental health, interpersonal concerns and behavioural concerns	5	✓ (n = 3)		✓ (n = 2)	[32]
Child Health Questionnaire (CHQ)	Measure HRQoL	General health perceptions, physical functioning, role/social physical functioning, bodily pain, role/social emotional functioning, role/social behavioural functioning, parent impact-time, parent impact-emotion, self-esteem, mental health, behaviour, family activities, family cohesion, change in health	14	✓ (n = 1)	✓ (n = 7)	✓ (n = 3)	[37]
Diabetes Stress Questionnaire	Measures Diabetes stressors	Distress-Worry, Peer Stress, Adverse-Personal Effects, Parental Stress, Hyperglycemia, Self-Care, Diet, and Hypoglycemia	8	✓ (n = 5)	✓ (n = 1)	✓ (n = 3)	[42]
Monitoring Individual Needs in Diabetes Youth Questionnaire (MY-Q)	Measure HRQoL in adolescents with Diabetes	General QoL, the teenagers’ social life (friends, family, and school), diabetes management (worries, treatment barriers, self-efficacy and satisfaction, and problematic eating), and emotional well-being	4	✓ (n = 1)	✓ (n = 4)	✓ (n = 1)	[36]
Patient-Reported Outcomes Measurement Information System (PROMIS) peadiatric profile -25	Measure that evaluates and monitors physical, mental, and social health in adolescents and children (HRQoL)	anxiety, depressive symptoms, fatigue, pain interference, physical function-mobility, and peer relationships as well as a single pain intensity item	7	✓ (n = 2)	✓ (n = 1)	✓ (n = 4)	[33, 45, 50]
Quality of Life Profile for Chronic Diseases (PLC)	Measures HRQoL in chronic patients	Capacity, positive mood, negative mood, ability to relax and enjoy, sense of belonging to others, contact ability	6	✓ (n = 1)	✓ (n = 4)	✓ (n = 2)	[40]
Questions on Life Satisfaction (FLZ)	Measures subjective QoL	Friends/acquaintances, leisure, general health, income, occupation, housing, family life, partner relationship/sexuality, physical, ability to relax, energy, mobility, vision & hearing, freedom from anxiety, freedom from pain, independence from help	16	✓ (n = 1)	✓ (n = 7)	✓ (n = 5)	[40]
Short Form 36 Health Survey (SF-36)	Measure HRQoL	Limitations in physical activities because of health problems Limitations in social activities because of physical or emotional problems Limitations in usual role activities because of physical health problems Bodily pain General mental health (psychological distress and well-being) Limitations in usual role activities because of emotional problems Vitality (energy and fatigue) General health perceptions	8	✓ (n = 3)	✓ (n = 1)	✓ (n = 5)	[40]

### Measuring constructs of mental health and mental well-being

With the exception of the WHO-5 well-being index [36, 37], all instruments measured domains associated with either mental health, mental well-being or both. For example, five of the mental well-being instruments are aimed at measuring different constructs related to mental well-being such as: resilience [41], hopefulness [51], self-esteem or sense of coherence [31] and spirituality [35]. These concepts refer to hedonic dimensions of mental well-being, i.e. are associated with ‘feeling well’. The exception is measuring *resilience* as a concept, which related to function or eudemonic well-being. This suggests that studies using instruments which measure singular constructs of mental well-being may be interested in understanding how hedonic (feeling well) indicators influence the general mental health and well-being of adolescents with a physical CC. On the other hand, it may be that eudemonic (functioning well) measures are being underrepresented or that these indicators are subsumed as subscales in HRQoL measures. However, each of these instruments were used only once, whereas the WHO-5 Well-being Index was used twice, indicating that instruments which include multiple domains of mental health and well-being may be preferable to instruments which measure singular constructs.

Additionally, seven of the instruments measured constructs that are detriments to mental health such as emotional and behavioural problems [38, 39, 43, 44, 46], symptoms of depression [30, 31, 37], symptoms of anxiety [30] and hopelessness [31]. These instruments measure negative feelings; except for the SDQ which also measures behaviours that can negatively affect mental health. The SDQ measure was used frequently across the studies suggesting that both emotional and behavioural risks to mental health are important considerations among adolescents living with a physical CC. Additionally, measures screening for depression among adolescents with a CC were used frequently. This is not surprising as adolescents with a physical CC are at risk of developing depression as a co-morbidity. The CESD scale is the only measure of depression in this review which measures a positive aspect of mental health (positive affect). Screening for depressive symptoms may help prevent onset of disorders.

The Diabetes Stress questionnaire is a disease specific instrument which measures mental health and mental well-being in adolescents living with diabetes. It is included in this category as it measures specific stressors related to living with diabetes, which may negatively impact mental health. Additionally, it includes the subscale of ‘self-care’ which is related to the eudemonic well-being construct of self-efficacy.

### Health-related quality of life and quality of life

The review identified various HRQoL and QoL instruments that were used as mental health and mental well-being measures. HRQoL and QoL are often related to mental well-being measures as the social indicator’s movement in the 1950s, which pertained to quality of life, gave rise to the development of theoretically based and validated instruments of positive psychological functioning including a sense of well-being and hope [52]. This may explain why none of the HRQoL or QoL instruments can be categorised as having only mental health subscales. HRQoL has been developed into a multi-dimensional concept that includes domains related to physical, mental, emotional, and social functioning. It goes beyond direct measures of population health, life expectancy, and causes of death, and focuses on the impact health status has on quality of life [53].

From Table 2, seven of the HRQoL include both mental health and mental well-being subscales, namely: Patient-Reported Outcomes Measurement Information System (PROMIS) paediatric profile -25 [33, 46, 50]; Child Health Questionnaire [37]; EQ-5D-Y [43]; Monitoring Individual Needs in Diabetes Youth Questionnaire (MY-Q) [36]; Quality of Life Profile for Chronic Diseases (PLC) [40]; Paediatric Quality of Life Inventory Diabetes Module (PedsQL-DMTM) [42]; and the Short Form 36 Health Survey (SF-36) [40]. Additionally, the Questions on Life Satisfaction (FLZ) was identified as a QoL measure [40].

In this category the PROMIS instrument paediatric profile-25 [30, 31, 37] was used frequently. Additionally, PROMIS, EQ-5D-Y and CHQ are the only instruments in this category designed to measure HRQoL in general child and adolescent populations and those living with a physical CC or illness. This may allow for comparisons to be made between general child/adolescent populations and those living with a CC. In comparison to the CHQ, the PROMIS measure has fewer subscales. However, in these subscales’ mental health (anxiety, depression) and physical well-being are emphasized, whereas the CHQ seem to emphasize the mental well-being subscales. Similarly, the EQ-5D-Y focuses more on physical aspects of well-being. The PROMIS may be used more frequently to provide information regarding the prevalence of depressive and anxiety symptoms in relation to physical functioning.

Similarly, the MY-Q and PedsQL-DMTM are both disease specific measures designed for child and adolescents living with diabetes. The four subscales of the MY-Q mostly emphasize both hedonic and eudemonic mental well-being concepts such as social and emotional well-being, self-efficacy satisfaction and general QoL. However, the ‘diabetes management’ subscale includes aspects which also relates to mental health (worries) and physical well-being (problematic eating and treatment barriers). The PedsQL-DMTM also has four subscales which represent mental health, well-being and physical well-being as it relates to living with diabetes specifically.

The SF-36 is a validated and well-researched measure of QoL in adult populations. In this review it was used in a comparison study with the PLC and FLZ instruments on a sample of adolescents and adults living with cystic fibrosis. Both the PLC and FLZ instruments were developed in Germany to measure QoL in the general population with the PLC being designed specifically for those with a chronic disease. Compared to the other instruments in this review, these may be less suited to use with adolescent populations.

Additionally, six of the HRQoL and three QoL measures are categorized as mental well-being measures. These are the Paediatric Quality of Life Inventory (PedsQL) [34, 36, 50], The KIDSREEN questionnaires [34, 43, 46–48], Cystic Fibrosis Questionnaire (CFQ) [51],Vécu et Santé Perçue des Adolescents (VSP-A) [49], WHO Quality of Life BREF [32]; Cerebral Palsy Quality of Life Questionnaire-Teen [34, 46]; and the Quality of Life in Short Stature Youth (QoLiSSY) [48].

The KIDSCREEN questionnaires, PedsQL and VSP-A were specifically developed to measure HRQoL in adolescent populations. The three versions of the KIDSCREEN questionnaires provide some flexibility for researchers who may choose to use the questionnaire based on completion time. For example, for younger adolescents it may be more appropriate to use the KIDSCREEN-10 (5 min) or KIDSCREEN-27 (10–15 min) to ensure they do not get fatigued while filling out the questionnaire. The KIDSCREEN-52 (15–25 min) may be more appropriate for older adolescents. Additionally, the KIDSCREEN questionnaires were designed to measure physical well-being and emphasized both hedonic and eudemonic dimensions of mental well-being. Similarly, the Peds-QL measures physical well-being and hedonic and eudemonic well-being. The VSP-A is a French instrument which has similar well-being subscales as the KIDSCREEN and Peds-QL. However, it also includes ‘vitality’ and ‘relationship with medical staff’ which may be useful to assess in adolescents who have chronic conditions.

The CFQ is a disease specific instrument that can be used a measure of mental well-being among adolescent and adult populations diagnosed with cystic fibrosis. The Cerebral Palsy Quality of Life Questionnaire-Teen and QoLiSSY instruments are both age and disease specific measures of well-being. Unlike other instruments included in this review, the QoLiSSY includes a specific ‘coping’ subscale. Coping is often listed as an important indicator of eudemonic well-being as it speaks to one’s ability to overcome challenges and improve resilience. It may be that ‘coping’ items are included within other subscales in the instruments such as the ‘individual’ subscale in the CYRM-28 measure or the ‘psychological well-being’ scale in the KIDSCREEN.

### Healthcare needs instruments

The Healthcare Needs Scale for Youth with Congenital Heart Disease (HNS-CHD) measures the transitional healthcare needs of adolescents living with a congenital heart disease. The questionnaire on health needs for adolescents [32] measures the healthcare needs of adolescents and was used to establish the concurrent validity of the HNS-CHD [41]. These instruments measure mental health and mental well-being by looking at the healthcare needs of adolescents which allow for early identification of mental health problems. However, not much information about these instruments is available outside of this initial study [41].

### Physical well-being subscales

We found that 21 of the instruments included one or more subscales related to physical well-being. As shown in Table 2, some of the physical well-being subscales may overlap with the mental health and mental well-being subscales. For example, the Center for Epidemiological Studies Depression (CESD) questionnaire includes 4 subscales aimed at measuring symptoms of depression. One of the subscales, ‘somatic complaints’ relates to physical symptoms of depression. However, it is also related to the individual’s physical well-being. Similarly, the Short Form Health 36 Survey (SF-36) includes subscales like ‘Limitations in social activities because of physical or emotional problems’, which again relates to both physical well-being and mental health (i.e. emotional state has a negative effect on functioning). This is not surprising as mental health, as it relates to mental illness considers somatic symptoms or functioning capabilities as an indicator to diagnose and assess the severity of an individual’s mental illness.

## Discussion

The increase in HRQoL studies on people living with a CC, indicates a shift to include social and psychological dimensions of health in biomedical research [9]. Our review confirmed that HRQoL is considered as a useful measure for assessing physical and psychosocial well-being among adolescents living with a CC. However, there are concerns that measures of HRQoL may focus more on physical health domains and therefore may not be appropriate for measuring HRQoL in people with mental health problems [54]. However, as shown in Table 3, and confirmed by Bech et al. [55], HRQoL instruments have more domains relating to mental well-being than physical well-being. Indeed, we found that many of the *other* instruments measuring mental well-being also included measures of HRQoL or QoL.

Additionally, adolescents in these studies were not diagnosed with any mental health problems. Therefore, it may be that these HRQoL instruments are useful for assessing the mental well-being of adolescents with a CC before they develop or are diagnosed with mental health problems. The PedsQL-DMTM was the only HRQoL instrument which measured a mental health domain (worry). This suggests that while mental well-being may be a key and necessary feature in HRQoL research, it may be that mental health is either underrepresented as Connel et al. [56] argue, or it may be that measuring mental health is not considered useful in certain contexts. According to Bech [55], the mixture of distress and well-being items has become increasingly problematic as well-being is an important aspect of HRQoL while mental health measures are related to the stipulations of diagnostic manuals such as the DSM V and ICD-10. However, as seen from Table 3, instruments often include both mental health (distress) and mental well-being domains to decrease floor and ceiling effects. In their study, Bech et al. [55] show that despite containing ‘pure’ well-being items, the WHO-Five measure was found to be more sensitive and had lower ceiling effects in comparison to the SF-36 mental health (distress) scales. This reflects a conceptual problem related to the psychometrics of measuring mental health—that is, to what extent is the absence of mental disorder symptoms equal to a high degree of psychological well-being? Nevertheless, this highlights the importance of conceptualising mental health and mental well-being as it will determine which instruments are most appropriate to use.

Sawyer et al. discussed issues relating to HRQoL measures as there are discrepancies between parental proxy reports and adolescent’s self-reports [9]. We found that ten of the included studies used self-report measures to measure HRQoL while five included both parent and adolescent reports. This suggests that self-report measures with adolescents living with a CC may be preferred to measure HRQoL. Of the five studies using HRQoL self- and parent reports, three [33, 34, 46] reported that there were discrepancies between the adolescents and parents, which were discussed as being consistent with previous studies. According to the KIDSCREEN group [57], there are issues regarding discrepancies between parent and adolescent reports, yet sometimes proxy reports are necessary for additional information or when the adolescent is unable to respond. Additionally, parent perspectives are important as they contribute to health-care decision making [57]. However, if there are any discrepancies between the child/adolescent report, the adolescent should be considered the preferred respondent [9, 37, 42].

Additionally, the choice of disease-specific or generic instruments should be considered as generic instruments facilitate comparisons between adolescents with different conditions and population norms whereas disease specific instruments can measure differential effects related to a specific disorder [56]. Our findings show that of the 31 instruments, 7 were disease specific (of which three focused on diabetes). Previously only a few studies have compared HRQoL across adolescents with different disorders as most studies focus on adolescent populations with a specific disorder. In our review we found three studies which involved samples of adolescents with different disorders. While disease specific studies are useful in describing the psychological effects (i.e., coping, adjustment, mental health problems) of individual diseases and conditions on adolescents and their families, research on the similarities and differences between disorders or diseases could inform practice and policy [56]. Additionally, measures that transcend specific diseases and conditions may help us better understand how structural elements of paediatric and mainstream healthcare systems can facilitate or hinder transitions of care [56]. Furthermore, considering the context of resource limited countries, it may be more practical to make comparisons between features of different disorders or conditions to understand how best to maximise health resources, design sustainable intervention programmes and establish adolescent friendly services for adolescents living with a CC.

The KIDSCREEN, SDQ, Paediatric Quality of Life and PROMIS instruments were repeatedly used across the 22 studies and proven to be reliable and valid instruments. Unlike the other instruments mentioned here, the SDQ is the only ‘mental health’ measure as it is often used as an emotional and behavioural screening tool rather than a measure of HRQoL or QoL. This is not to say that the SDQ is the only mental health/mental well-being measure to use among adolescents living with a CC. Indeed, there are a variety of reliable and valid measures of mental well-being and mental health to be used in adolescent populations such as the Warwick-Edinburgh Mental Well-being Scale (WEMWBS) or the Beck Youth Inventory. However, as shown in this review it may be that HRQoL instruments are more preferable for use in adolescent populations with a CC as Sawyer et al. [9] argued. Additionally, instruments such as the SDQ and KIDSCREEN were specifically developed for adolescent populations, whereas the WEMWBS is designed for all ages.

## Limitations and future research

We conducted a comprehensive and systematic review of the literature using broad search terms and criteria to ensure inclusion of all relevant articles. Unlike previous studies in this field [18, 24] our review included both general mental health and mental well-being measures. However, some limitations are noted. While we tried to keep the criteria as broad as possible, we acknowledge that there are inherent issues related to the databases we have chosen which may have restricted our access to certain articles. For example, the chosen databases may have restricted access to psychological journals. Additionally, we gained access to these databases via our institution. As such, we only had access to articles based on our institutional access. Additionally, it should be noted that this review forms part of a larger review focused on assessing mental health measures used among adolescents [3]. Therefore, this review represents a snapshot of our main review. Assessing general mental health and mental well-being among adolescents living with a CC is important for both clinicians and policy makers. However, future research should focus on clearly conceptualising what mental health and well-being means to adolescents, especially those living with a CC. This is relevant as we previously mentioned that there is currently no agreed upon definition of mental health and the way a concept is defined has implications for how it is measured. As seen in this study, we found that many of the mental health/well-being instruments were HRQoL instruments. Future research should also establish the validity of HRQoL instruments as measures of mental health/well-being through comparing the convergent validity of mental health/well-being instruments.

## Conclusions

Many adolescent CC are not preventable. However, the potential mental co-morbidities which can result from living with a life-long condition can be prevented or modified to ensure optimal quality of life. As such, the findings from our review reflect previous research trends suggesting that HRQoL measures seem to be more useful in measuring mental health and/or mental well-being among adolescents living with a CC as this allows for an all-round assessment of both physical, psychological and social outcomes. Measures such as the KIDSCREEN, SDQ and Paediatric Quality of Life scales are shown to be useful and valid measures to assess mental health and well-being among adolescents living with a CC in both developed and developing countries. However, all the instruments included in this study were developed in high income countries and then adapted for use in LMICs. While these instruments were useful, we would suggest that more instruments be developed in LMICs as this may provide us with more insight into which constructs of mental health/mental well-being and health are important to adolescents living in this context. Furthermore, such assessments may help researchers, policy makers and health professionals better understand the complex issues experienced by adolescents living with a CC in resource-limited settings. We recommend more research to compare adolescents with different CC, especially in LMICs, which will inform the development of new frameworks for healthcare systems that will [better] support the healthy development of adolescents living with a CC as they transition to adult life.

## Data Availability

The datasets generated and/or analysed during the current study are not publicly available due to the ongoing nature of the study but are available from the corresponding author on reasonable request.

## Abbreviations

CC: Chronic Condition
CDI: Child Depression Inventory
CFQ: Cystic Fibrosis Questionnaire
CHQ: Child Health Questionnaire
CYRM-28: Child Youth Resilience Measurement
FLZ: Questions on Life Satisfaction
HIC: High Income Countries
HOPES: Hunter Opinions and Personal Expectations Scale
HRQoL: Health Related Quality of Life
LMICs: Low- and Middle-Income Countries
MY-Q: Monitoring Individual Needs in Diabetes Youth Questionnaire
PedsQL-DMTM: Paediatric Quality of Life Inventory Diabetes Module
PLC: Quality of Life Profile for Chronic Diseases
PROMIS: Patient-Reported Outcomes Measurement Information System
QoL: Quality of Life
SDQ: Strengths and Difficulties Questionnaire
SF-36: Short Form 36 Health Survey
